# Association Between the Naples Score and Recurrence in Patients With Atrial Fibrillation Undergoing Radiofrequency Catheter Ablation

**DOI:** 10.1155/cdr/1400368

**Published:** 2025-12-19

**Authors:** Zhen Wang, Yilin Qu, Hua Wang, Chunxiao Wang

**Affiliations:** ^1^ Department of Cardiology, Yantai Yuhuangding Hospital, Qingdao University, Yantai, China, qdu.edu.cn; ^2^ Department of Medical Records, Yantai Yuhuangding Hospital, Qingdao University, Yantai, China, qdu.edu.cn

**Keywords:** atrial fibrillation, Naples Score, radiofrequency catheter ablation, recurrence

## Abstract

**Purpose:**

To explore the relationship between the Naples Score and the recurrence of atrial fibrillation (AF) following radiofrequency catheter ablation (RFCA) in patients.

**Methods:**

We conducted a retrospective analysis of 719 patients with AF who underwent radiofrequency catheter RFCA between April 2019 and October 2024 at Yantai Yuhuangding Hospital of Qingdao University. We utilized Cox multifactorial regression analysis and Kaplan–Meier survival curves to evaluate the relationship between the Naples Score and the recurrence of AF following RFCA. Additionally, a receiver operating characteristic (ROC) curve was generated to assess the predictive value of the Naples Score for recurrence.

**Results:**

After follow‐up, 156 (21.7%) AF patients occurred recurrence. A significant difference in recurrence rates was observed among patients with varying Naples Scores (14.0% vs. 20.8% vs. 34.2%, *p* < 0.001). The Cox multifactorial regression analysis indicated that the Naples Score was an independent risk factor for recurrence following RFCA of AF (hazard ratio [HR] = 1.28, *p* < 0.001). The postoperative recurrence rate was significantly higher in patients with elevated Naples Scores compared with those with lower scores (HR = 2.25, *p* < 0.001). Kaplan–Meier survival analysis revealed significant differences in recurrence rates among patients with different Naples Scores (Log − rank *p* < 0.001). ROC curve analysis demonstrated the predictive value of the Naples Score for recurrence after RFCA in AF, with an AUC of 0.62 (95% confidence interval [CI]: 0.57–0.66, *p* < 0.001).

**Conclusion:**

Naples Score was identified as an independent risk factor for recurrence following RFCA of AF.

## 1. Introduction

Atrial fibrillation (AF) is one of the most prevalent heart conditions. The prevalence of AF is projected to double in the coming decades due to an aging population, a rising burden of comorbidities, increased awareness, and advancements in detection technologies. AF is linked to a variety of serious adverse events. Patients with AF experience high rates of hospitalization and associated complications. Furthermore, individuals with AF have a fourfold to fivefold increased relative risk of developing heart failure compared with those without AF [1]. AF is also linked to increased mortality rates. In 2017, AF accounted for over 250,000 deaths worldwide, with an age‐standardized mortality rate of 4.0 per 100,000 individuals [2]. AF is associated with a twofold increased risk of all‐cause mortality and cardiovascular mortality compared with sinus rhythm (SR) [1, 3].

Current AF treatment remains suboptimal, not only due to inadequate interventional and pharmacological approaches but also because of an incomplete understanding of the mechanisms that promote local atrial and systemic AF in relation to various risk factors. In most patients, AF is a manifestation of atrial remodeling caused by a range of comorbidities and risk factors [4]. Inflammation is essential for eliminating the causes of cellular damage and facilitating its clearance; however, it is increasingly linked to AF. Mediators of the inflammatory response can modify atrial electrophysiology and structural substrates, resulting in heightened susceptibility to AF [5].

The Naples Score is a comprehensive prognostic model that integrates inflammatory and nutritional markers, including serum albumin, total cholesterol (TC), the neutrophil‐to‐lymphocyte ratio (NLR), and the lymphocyte‐to‐monocyte ratio (LMR). Previous studies have demonstrated that the Naples Score is significantly associated with the prognosis of patients with tumors [6–8]. NLR has been shown to be significantly associated with recurrence after radiofrequency catheter ablation (RFCA) of AF [9]. To date, the Naples Score has not been utilized to assess outcomes after RFCA in patients with AF. The purpose of this study is to evaluate the impact of the Naples Score on the recurrence of AF after RFCA.

## 2. Population and Methods

**Figure 1:**
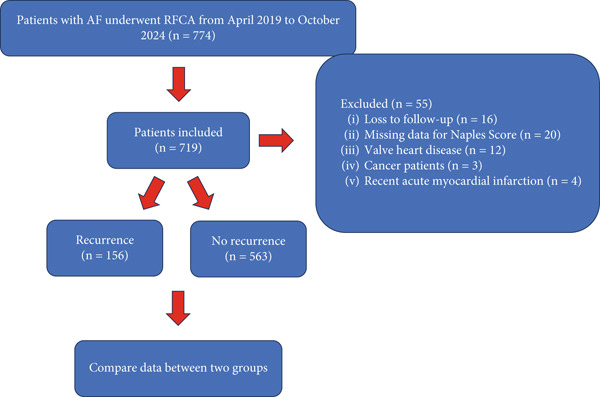
The flowchart of our project.

The flowchart of our study is presented in Figure 1. We retrospectively analyzed 719 patients with AF who underwent RFCA between April 2019 and October 2024 at Yantai Yuhuangding Hospital of Qingdao University. paroxysmal AF (PAF) was defined as episodes lasting less than 7 days, whereas persistent AF was defined as episodes lasting 7 days or more. All patients had relevant blood parameters collected within 24 h of admission, including a complete blood count, biochemical tests, and assessments of coagulation function. All patients underwent transthoracic echocardiography upon admission to assess left atrial diameter (LAD), left ventricular end‐diastolic diameter (LVEDD), and left ventricular ejection fraction (LVEF). Additionally, all patients were required to undergo transthoracic echocardiography prior to radiofrequency ablation to determine the presence of left atrial thrombosis. We excluded patients based on the following criteria [1] failure to follow up; [2] missing information regarding the Naples Score; [3] valvular heart disease; [4] recent history of myocardial infarction; and [5] previous history of tumors. The study adhered to the Declaration of Helsinki and received approval from the Institutional Review Committee of Yantai Yuhuangding Hospital of Qingdao University.

**Figure 2:**
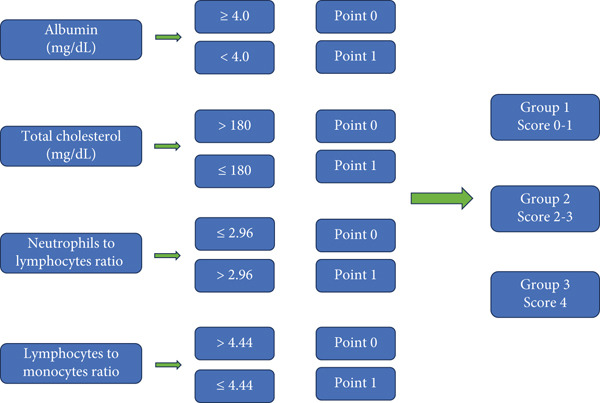
Naples Score categories and groupings.

Naples Score was calculated using four parameters: TC, albumin, the LMR, and the NLR. A scoring system was established based on the following criteria: a score of 0 was assigned if albumin was ≥ 4.0 mg/dL, TC was > 180 mg/dL, NLR was ≤ 2.96, and LMR was > 4.44. A score of 1 was assigned if any of these conditions were not met. Patients were categorized into three groups according to their Naples Score: patients with scores of 0–1 were placed in Group 1, those with scores of 2–3 were placed in Group 2, and patients with a score of 4 were placed in Group 3. See Figure 2.

### 2.1. Catheter Ablation

The specific ablation strategy can be referred to in our previously published articles [10]. All patients ultimately regained SR and were monitored for 30 min, with no recurrence of arrhythmia. Pericardial tamponade occurred in seven patients (0.9%) during radiofrequency ablation, and symptoms improved after the drainage of pericardial effusion. Additionally, 10 patients (1.4%) developed pseudoaneurysms during femoral vein puncture.

### 2.2. Follow‐Up

All patients received rivaroxaban treatment for a minimum of 3 months following the ablation procedure to prevent thrombosis. Clinical follow‐up included outpatient visits, which featured 24‐h Holter monitoring or 12‐lead ECG monitoring at 3, 6, and 12 months postablation. Subsequently, monitoring was conducted every 6 months. All patients underwent either 12‐lead ECG or 24‐h. Holter monitoring. recurrence was defined as AF, atrial flutter (AFL), or atrial tachycardia (AT) lasting more than 30 s. Early recurrence (ER) was defined as a recurrence of AF lasting 30 s or more within the first 3 months of follow‐up after RFCA. During the follow‐up period, four patients experienced cerebral ischemic strokes (CIS), which were later confirmed to be unrelated to AF. One patient developed a cerebral hemorrhage, which was confirmed by cerebral arteriography to be due to a ruptured cerebral aneurysm. All patients survived, and no deaths occurred.

### 2.3. Statistical Analyses

Statistical analysis and plotting were performed using SPSS 27.0 version, GraphPad Prism 8.0 version, and Z‐Stats (https://www.medsta.cn/). The Kolmogorov–Smirnov test was employed to assess whether the measurement data followed a normal distribution. Data that did not conform to a normal distribution were reported as median and interquartile range [M (IQR)], with group comparisons made using the Mann–Whitney nonparametric test. Categorical data were expressed as percentage component ratios (*n*, %), and group comparisons were performed using the *χ* ^2^ test. Cox multifactorial regression analysis was utilized to identify risk factors associated with recurrence after AF ablation. ROC curves were generated to evaluate the predictive value of risk factors for postoperative recurrence of AF. The DeLong test was applied to compare the areas under different ROC curves. Kaplan–Meier survival analysis was conducted to compare recurrence rates after RFCA of atrial AF across different Naples Score groups. A two‐sided *p* value < 0.05 was considered statistically significant.

## 3. Results

### 3.1. Baseline Clinical Data

Significant differences were observed among the various Naples score groups regarding AF duration, AF type, CHA_2_DS_2_‐VASc score, and medical history, including DM, CHD, CHF. Additionally, differences were noted in LAD, LVEDD, LVEF, glucose levels, TC levels, B‐type natriuretic peptide (BNP) levels, leukocyte counts, neutrophil counts, lymphocyte counts, the proportion of ablation to SR, the proportion of ER, and the overall recurrence rate (*p* < 0.05). See Table 1.

**Table 1:** Comparison of clinical data among different Naples Score groups.

**Variable**	**Group 1** **(** **n** = 292**)**	**Group 2** **(** **n** = 231**)**	**Group 3** **(** **n** = 196**)**	**p** **value**
Age (years)	61.00 (53.00, 67.00)	64.00 (58.00, 69.00)	66.00 (60.00, 73.00)	0.259
Gender (*n*, %)				0.739
Female	155 (53.10)	79 (34.20)	71 (36.20)	
Male	137 (46.90)	152 (65.80)	125 (63.80)	
AF duration (months)	24.00 (6.00, 57.00)	24.00 (5.00, 60.00)	24.00 (6.00, 60.00)	< 0.001
AF Type (*n*, %)				< 0.001
PeAF	134 (45.90)	105 (45.50)	121 (61.70)	
PAF	158 (54.10)	126 (54.50)	75 (38.30)	
CHA_2_DS_2_‐VASc Score	2.00 (1.00, 3.00)	2.00 (1.00, 3.00)	2.00 (1.00, 4.00)	0.014
Comorbidity				
AFL (*n*, %)				0.353
Yes	26 (8.90)	22 (9.50)	21 (10.70)	
No	266 (91.10)	209 (90.50)	175 (89.30)	
Hypertension (*n*, %)				0.096
Yes	164 (56.20)	115 (49.80)	126 (64.30)	
No	128 (43.80)	116 (50.20)	70 (35.70)	
DM (*n*, %)				0.035
Yes	35 (12.00)	35 (15.20)	47 (24.00)	
No	257 (88.00)	196 (84.80)	149 (76.00)	
CHD (*n*, %)				0.004
Yes	24 (8.20)	44 (19.00)	41 (20.90)	
No	268 (91.80)	187 (81.00)	155 (79.10)	
CHF (*n*, %)				< 0.001
Yes	19 (6.50)	25 (10.80)	34 (17.30)	
No	273 (93.50)	206 (89.20)	162 (82.70)	
COPD (*n*, %)				0.054
Yes	—	4 (1.70)	7 (3.60)	
No	233 (100.00)	227 (98.30)	189 (96.40)	
CIS (*n*, %)				0.311
Yes	21 (7.20)	35 (15.20)	33 (16.80)	
No	271 (92.80)	196 (84.80)	163 (83.20)	
Imaging factors				
LAD (mm)	40.00 (36.00, 44.00)	41.00 (37.00, 45.00)	42.00 (39.00, 46.00)	< 0.001
LVEF (mm)	65.00 (61.00, 68.00)	64.00 (59.00, 67.00)	63.00 (57.00, 67.00)	0.021
LVEDD (mm)	45.00 (42.00, 48.00)	46.00 (43.00, 50.00)	46.00 (42.00, 51.00)	< 0.001
Laboratory index				
AST (U/L)	22.00 (18.00, 25.75)	22.00 (18.00, 30.00)	21.00 (17.00, 27.00)	0.088
ALT (U/L)	21.00 (16.00, 28.00)	22.00 (15.00, 33.00)	20.00 (15.00, 31.00)	0.366
Glucose (mmol/L)	5.33 (4.83, 5.96)	5.29 (4.90, 6.09)	5.37 (4.84, 6.76)	< 0.001
SCr (*μ*mol/L)	63.00 (53.00, 73.00)	67.00 (57.00, 79.00)	67.00 (57.00, 79.00)	0.838
SUA (*μ*mol/L)	344.00 (297.00, 409.00)	355.00 (292.00, 416.00)	359.00 (289.25, 405.75)	0.808
Albumin (g/L)	41.59 (40.15, 43.55)	40.68 (38.45, 42.20)	38.44 (36.54, 39.63)	0.050
TG (mmol/L)	1.35 (0.93, 1.78)	1.09 (0.85, 1.54)	0.98 (0.75, 1.25)	0.434
TC (mmol/L)	5.22 (4.76, 5.80)	4.29 (3.70, 5.10)	3.92 (3.39, 4.42)	< 0.001
LDL‐C (mmol/L)	3.42 (2.88, 3.80)	2.59 (2.06, 3.27)	2.37 (1.88, 2.76)	0.898
HDL‐C (mmol/L)	1.31 (1.15, 1.50)	1.17 (1.04, 1.37)	1.14 (0.97, 1.35)	0.057
HCY (mg/L)	12.35 (10.50, 13.98)	12.70 (10.70, 15.60)	12.55 (10.50, 15.18)	0.116
BNP (pg/mL)	70.79 (31.49, 166.32)	101.59 (53.76, 192.57)	161.87 (70.55, 338.70)	0.001
D‐dimer (mg/L)	0.55 (0.36, 0.70)	0.59 (0.48, 0.80)	0.65 (0.50, 0.94)	0.658
Hemoglobin (×10^9^/L)	147.00 (134.00, 159.00)	146.00 (138.00, 156.00)	143.50 (131.25, 156.75)	0.277
Leukocyte (×10^9^/L)	6.06 (5.03, 7.05)	6.37 (5.35, 7.45)	6.75 (5.41, 8.21)	0.013
Platelet (×10^9^/L)	226.00 (197.00, 260.00)	215.00 (186.00, 249.00)	205.00 (173.00, 250.00)	0.713
Neutrophil (×10^9^/L)	3.44 (2.70, 4.12)	3.79 (3.01, 4.75)	4.50 (3.35, 5.78)	< 0.001
Lymphocyte (×10^9^/L)	1.99 (1.62, 2.40)	1.72 (1.44, 2.12)	1.44 (1.13, 1.74)	0.004
Monocyte (×10^9^/L)	0.42 (0.34, 0.52)	0.51 (0.42, 0.61)	0.49 (0.39, 0.61)	0.111
Naples Score	1.00 (0.00, 1.00)	2.00 (2.00, 2.00)	3.00 (3.00, 4.00)	< 0.001
Ablation procedure				
Left atrial roof (*n*, %)				0.060
Yes	70 (24.00)	41 (17.70)	56 (28.60)	
No	222 (76.00)	190 (82.30)	140 (71.40)	
Left atrial anterior wall (*n*, %)				0.104
Yes	8 (2.70)	6 (2.60)	12 (6.10)	
No	284 (97.30)	225 (97.40)	184 (93.90)	
Tricuspid isthmus (*n*, %)				0.166
Yes	63 (21.60)	67 (29.00)	48 (24.50)	
No	229 (78.40)	164 (71.00)	148 (75.50)	
Mitral isthmus (*n*, %)				0.055
Yes	23 (7.90)	12 (5.20)	37 (18.90)	
No	269 (92.10)	219 (94.80)	159 (81.10)	
Ablation to SR (*n*, %)				< 0.001
Yes	174 (59.60)	133 (57.60)	88 (44.90)	
No	118 (40.40)	98 (52.40)	108 (55.10)	
Ablation time (minutes)	125.00 (100.00, 155.00)	130.00 (105.00, 170.00)	135.00 (114.00, 180.00)	< 0.001
ER (*n*, %)				< 0.001
Yes	69 (23.60)	39 (16.90)	34 (17.30)	
NO	223 (76.40)	192 (83.10)	162 (82.70)	
Recurrence (*n*, %)				< 0.001
Yes	41 (14.00)	48 (20.80)	67 (34.20)	
NO	251 (86.00)	183 (79.20)	129 (65.80)	

Abbreviation: AF, atrial fibrillation; AFL, atrial flutter; ALT, alanine aminotransferase; AST, aspartate aminotransferase; BNP, brain natriuretic peptide; CHD, coronary heart disease; CHF, chronic heart failure; CIS, cerebral ischemic stroke; COPD, chronic obstructive pulmonary disease; DM, diabetes mellitus; ER, early recurrence; HCY, homocysteine; HDL‐C, high‐density cholesterol; LAD, left atrial diameter; LDL‐C, low‐density cholesterol; LVEDD, left ventricular end‐diastolic diameter; LVEF, left ventricular ejection fraction; PAF, paroxysmal atrial fibrillation; PeAF, persistent atrial fibrillation; Scr, serum creatinine; SR, sinus rhythm; SUA, serum uric acid; TC, total cholesterol; TG, triglyceride.

There were significant differences between the recurrence and no‐recurrence groups in terms of AF duration, AF type, CHA_2_DS_2_‐VASc score, history of DM, history of CHD, history of CHF, LAD, LVEDD, LVEF, glucose levels, albumin levels, BNP levels, neutrophil levels, lymphocyte levels, leukocyte levels, Naples Score, proportion of ablation to SR, ablation time, and the proportion of ER (*p* < 0.05). See Table 2.

**Table 2:** Comparison of clinical data between recurrence and nonrecurrence groups.

**Variable**	**Total** **(** **n** = 719**)**	**Recurrence** **(** **n** = 156**)**	**No-recurrence** **(** **n** = 563**)**	**p** **value**
Age (years)	63.00 (57.00, 69.00)	64.00 (57.25, 70.00)	63.00 (57.00, 69.00)	0.104
Gender (n, %)				0.738
Female	305 (42.40)	68 (43.60)	237 (42.10)	
Male	414 (57.60)	88 (56.40)	326 (57.90)	
AF duration (months)	24.00 (6.00, 60.00)	36.00 (12.00, 84.00)	14.00 (4.00, 48.00)	< 0.001
AF Type (*n*, %)				< 0.001
PeAF	360 (50.10)	124 (79.50)	236 (41.90)	
PAF	359 (49.90)	32 (20.50)	327 (58.10)	
CHA_2_DS_2_‐VASc Score	2.00 (1.00, 3.00)	2.00 (1.00, 3.00)	2.00 (1.00, 3.00)	0.008
Comorbidity				
AFL (*n*, %)				0.352
Yes	69 (9.60)	18 (11.50)	51 (9.10)	
No	650 (90.40)	138 (88.50)	512 (90.90)	
Hypertension (*n*, %)				0.096
Yes	405 (56.30)	97 (62.20)	308 (54.70)	
No	314 (43.70)	59 (37.80)	255 (45.30)	
DM (*n*, %)				0.035
Yes	117 (16.30)	34 (21.80)	83 (14.70)	
No	602 (83.70)	122 (78.20)	480 (85.30)	
CHD (*n*, %)				0.004
Yes	109 (15.20)	35 (22.40)	74 (13.10)	
No	610 (87.80)	121 (77.60)	489 (86.90)	
CHF (*n*, %)				< 0.001
Yes	78 (10.80)	30 (19.20)	48 (8.50)	
No	641 (89.20)	126 (80.80)	515 (91.50)	
COPD (*n*, %)				0.054
Yes	11 (1.50)	5 (3.20)	6 (1.10)	
No	708 (98.50)	151 (96.80)	557 (98.90)	
CIS (*n*, %)				0.311
Yes	89 (12.40)	23 (14.70)	66 (11.70)	
No	630 (87.60)	133 (85.30)	497 (88.30)	
Imaging factors				
LAD (mm)	41.00 (37.00, 45.00)	45.00 (40.78, 49.00)	40.00 (36.00, 44.00)	< 0.001
LVEF (mm)	64.00 (60.00, 67.00)	62.00 (57.00, 65.00)	65.00 (61.00, 68.00)	< 0.001
LVEDD (mm)	46 (42.90, 49.00)	47.00 (43.00, 51.88)	46.00 (42.00, 49.00)	0.008
Laboratory index				
AST (U/L)	22.00 (18.00, 27.00)	21.50 (17.25, 28.00)	22.00 (18.00, 26.00)	0.789
ALT (U/L)	21.00 (15.00, 30.00)	20.50 (15.25, 31.00)	21.00 (15.00, 30.00)	0.997
Glucose (mmol/L)	5.31 (4.85, 6.09)	5.59 (4.90, 6.75)	5.27 (4.85, 5.99)	0.001
SCr (*μ*mol/L)	65.00 (55.00, 76.00)	66.00 (56.00, 77.00)	64.00 (55.00, 75.00)	0.351
SUA (*μ*mol/L)	351.00 (295.00, 409.00)	359.00 (295.25, 412.50)	347.00 (293.00, 409.00)	0.591
Albumin (g/L)	40.54 (38.44, 42.29)	39.76 (38.15, 41.98)	40.73 (38.59, 42.46)	0.047
TG (mmol/L)	1.14 (0.85, 1.59)	1.04 (0.83, 1.59)	1.16 (0.86, 1.59)	0.181
TC (mmol/L)	4.58 (3.82, 5.37)	4.48 (3.79, 5.33)	4.60 (3.84, 5.39)	0.473
LDL‐C (mmol/L)	2.82 (2.20, 3.51)	2.82 (2.21, 3.47)	2.82 (2.20, 3.52)	0.696
HDL‐C (mmol/L)	1.23 (1.05, 1.43)	1.23 (1.06, 1.47)	1.23 (1.04, 1.42)	0.438
HCY (mg/L)	12.50 (10.50, 14.90)	12.50 (10.73, 15.40)	12.50 (10.50, 14.60)	0.383
BNP (pg/mL)	98.06 (44.16, 221.08)	153.57 (78.38, 281.34)	90.51 (39.86, 196.84)	<0.001
D‐dimer (mg/L)	0.59 (0.45, 0.78)	0.60 (0.47, 0.86)	0.58 (0.44, 0.76)	0.141
Hemoglobin (×10^9^/L)	146.00 (135.00, 157.00)	145.50 (133.25, 157.75)	146.00 (135.00, 157.00)	0.402
Leukocyte (×10^9^/L)	6.22 (5.21, 7.53)	6.57 (5.14, 8.03)	6.19 (5.21, 7.42)	0.047
Platelet (×10^9^/L)	217.00 (187.00, 256.00)	215.50 (182.25, 257.00)	217.00 (187.00, 255.00)	0.991
Neutrophil (×10^9^/L)	3.82 (2.99, 4.65)	4.22 (3.06, 5.32)	3.70 (2.96, 4.53)	0.002
Lymphocyte (×10^9^/L)	1.73 (1.42, 2.15)	1.63 (1.28, 2.01)	1.76 (1.45, 2.20)	0.003
Monocyte (×10^9^/L)	0.47 (0.37, 0.57)	0.47 (0.39, 0.62)	0.47 (0.37, 0.56)	0.306
Naples Score	2.00 (1.00, 3.00)	2.00 (2.00, 3.00)	2.00 (1.00, 3.00)	< 0.001
Naples Score (*n*, %)				< 0.001
Group 1	292 (40.60)	41 (26.30)	251 (44.60)	
Group 2	131 (32.10)	48 (30.80)	183 (32.50)	
Group 3	196 (27.30)	67 (42.90)	129 (22.90)	
Ablation procedure				
Left atrial roof (*n*, %)				0.104
Yes	167 (23.20)	45 (28.80)	122 (21.70)	
No	552 (76.80)	111 (71.20)	441 (88.30)	
Left atrial anterior wall (*n*, %)				0.060
Yes	26 (3.60)	9 (5.80)	17 (3.00)	
No	693 (96.40)	147 (94.20)	546 (97.00)	
Tricuspid isthmus (n, %)				0.165
Yes	178 (24.80)	32 (20.50)	146 (25.90)	
No	541 (75.20)	124 (79.50)	417 (74.10)	
Mitral isthmus (*n*, %)				0.055
Yes	72 (10.00)	22 (14.10)	50 (8.90)	
No	647 (90.00)	134 (85.90)	513 (91.10)	
Ablation to SR (*n*, %)				< 0.001
Yes	395 (54.90)	40 (25.60)	355 (63.10)	
No	324 (45.10)	116 (77.40)	208 (36.90)	
Ablation time (minutes)	130.00 (105.00, 170.00)	140.00 (120.00, 180.00)	125.00 (100.00, 165.00)	< 0.001
ER (*n*, %)				< 0.001
Yes	142 (19.70)	76 (48.70)	66 (11.70)	
NO	577 (80.30)	164 (70.4)	497 (88.30)	

Abbreviation: AF, atrial fibrillation; AFL, atrial flutter; ALT, alanine aminotransferase; AST, aspartate aminotransferase; BNP, brain natriuretic peptide; CHD, coronary heart disease; CHF, chronic heart failure; CIS, cerebral ischemic stroke; COPD, chronic obstructive pulmonary disease; DM, diabetes mellitus; ER, early recurrence; HCY, homocysteine; HDL‐C, high‐density cholesterol; LAD, left atrial diameter; LDL‐C, low‐density cholesterol; LVEDD, left ventricular end‐diastolic diameter; LVEF, left ventricular ejection fraction; PAF, paroxysmal atrial fibrillation; PeAF, persistent atrial fibrillation; Scr, serum creatinine; SR, sinus rhythm; SUA, serum uric acid; TC, total cholesterol; TG, triglyceride.

### 3.2. Relationship Between the Naples Score and AF Recurrence After RFCA

There was a significant difference in recurrence rates after RFCA of AF among patients with varying Naples Score groups (14.0% vs. 20.8% vs. 34.2%, *p* < 0.001). See Figure 3a.

Figure 3Comparison of recurrence rates among different Naples Score groups (a) and comparison of Naples Scores in recurrence and nonrecurrence groups (b).

**Figure:**
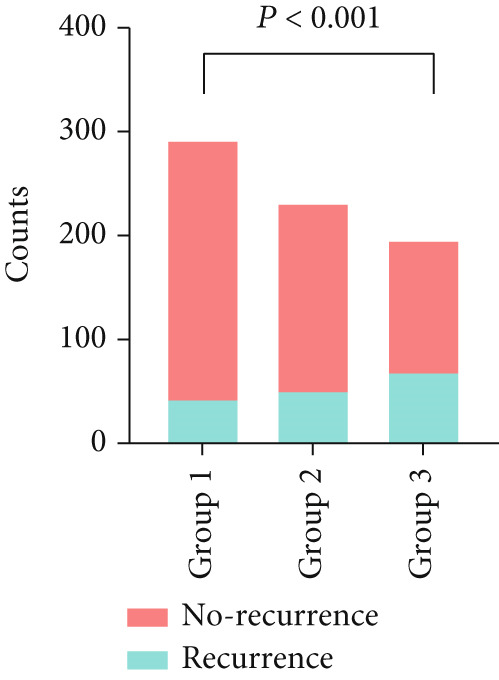
(a)

**Figure:**
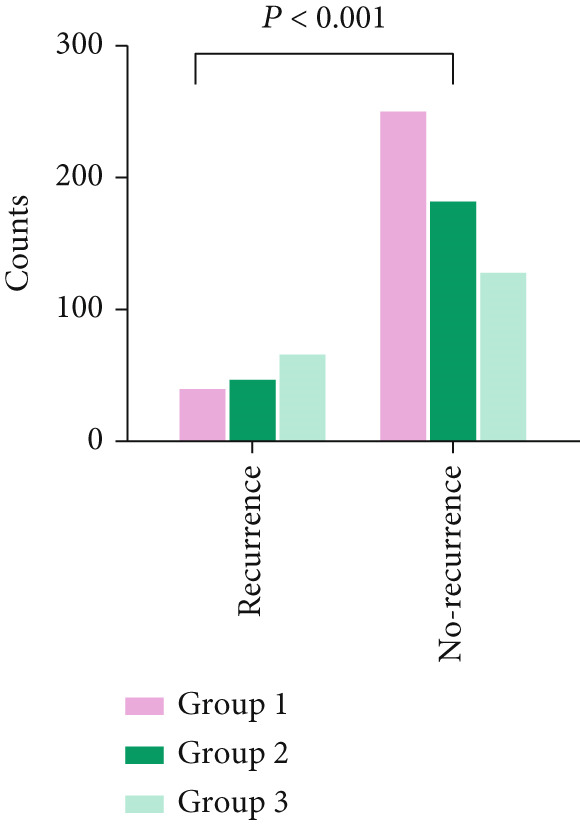
(b)

Naples Scores were significantly higher in the recurrence group compared with the no‐recurrence group (*p* < 0.001). See Figure 3b.

Univariate Cox regression analysis indicated that AF duration, AF type, history of DM, history of CHD, history of CHF, LAD, ER, ablation to SR, CHA_2_DS_2_‐VASc score, and Naples Score were associated with recurrence after RFCA. Multifactorial Cox regression analysis revealed that AF duration (HR = 1.00, *p* < 0.001), persistent AF (HR = 2.46, *p* = 0.01), LAD (HR = 1.07, *p* < 0.001), ER (HR = 4.67, *p* < 0.001), and Naples Score (HR = 1.28, *p* < 0.001) were independent risk factors for recurrence after RFCA for AF. See Table 3.

**Table 3:** Cox regression analysis of recurrence following RFCA.

**Variable**	**Univariate analysis**		**Multifactor analysis**	
	**HR (95% CI)**	**p** **value**	**HR (95% CI)**	**p** **value**
AF duration	1.00 (1.00–1.01)	< 0.001	1.00 (1.00–1.01)	< 0.001
AF type		0.010		0.010
PAF	Reference		Reference	
PeAF	6.50 (4.34–9.74)		2.46 (1.24–4.86)	
DM		0.034		
No	Reference			
Yes	1.51 (1.03–2.21)			
CHD		0.001		
No	Reference			
Yes	1.88 (1.29–2.74)			
CHF		< 0.001		
No	Reference			
Yes	2.87 (1.92–4.29)			
LAD	1.14 (1.11–1.18)	< 0.001	1.07 (1.04–1.11)	< 0.001
ER		< 0.001	4.67 (3.29–6.61)	< 0.001
No	Reference			
Yes	6.39 (4.65–8.79)			
Naples Score	1.31 (1.15–1.51)	< 0.001	1.28 (1.11–1.46)	< 0.001
Ablation to SR		< 0.001		
No	Reference			
Yes	0.16 (0.11–0.24)			
CHA_2_DS_2_‐VASc Score	1.12 (1.02–1.24)	0.018		

Abbreviation: AF, atrial fibrillation; CHD, coronary heart disease; CHF, chronic heart failure; DM, diabetes mellitus; ER, early recurrence; LAD, left atrial diameter; PAF, paroxysmal atrial fibrillation; PeAF, persistent atrial fibrillation; SR, sinus rhythm.

As shown in Table 4, the Naples Score was treated as a categorical variable in the multifactorial Cox regression analysis. The results confirmed that patients with a high Naples Score experienced a higher rate of postoperative recurrence compared with those with a low Naples Score (HR = 2.25, *p* < 0.001).

**Table 4:** Multivariate Cox regression analysis.

**Variable**	**HR (95% CI)**	**p** **value**
AF duration	1.00 (1.00–1.01)	< 0.001
AF type		0.013
PAF	Reference	
PeAF	2.39 (1.21–4.74)	
LAD	1.07 (1.04–1.11)	< 0.001
ER		< 0.001
No	Reference	
Yes	4.84 (3.34–6.88)	
Naples Score		
Group 1	Reference	
Group 2	1.63 (1.06–2.50)	0.026
Group 3	2.25 (1.51–3.36)	< 0.001

*Notes:* Naples Score was included in Cox regression analysis as a categorical variable.

Abbreviation: AF, atrial fibrillation; ER, early recurrence; LAD, left atrial diameter; PAF, paroxysmal atrial fibrillation; PeAF, persistent atrial fibrillation.

As illustrated in Table 5 and Figure 4, the ROC curve examined the relationship between the Naples Score and the recurrence of AF. The area under the curve (AUC) was 0.62 (95% CI: 0.57–0.66, *p* < 0.001). The predictive value derived from the model of established risk factors, which included AF duration, AF type, LAD, and ER, was 0.78 (95% CI: 0.74–0.83, *p* < 0.001). However, the predictive value of the established risk factor model (including AF duration, AF type, LAD, ER, and Naples Score) was 0.81 (95% CI: 0.77–0.85, *p* < 0.001). The DeLong test indicated a significant difference between the two model groups (*Z* = 3.37, *p* < 0.001).

**Table 5:** ROC curve of risk factors for recurrence following RFCA of AF.

**Variable**	**AUC**	**95% CI**	**Sensitivity**	**Specificity**	**p** **value**	
LAD	0.71	0.66–0.76	0.63	0.71	< 0.001	
AF duration	0.63	0.59–0.68	0.50	0.71	< 0.001	
AF type	0.69	0.65–0.73	0.58	0.79	< 0.001	
ER	0.68	0.64–0.73	0.88	0.49	< 0.001	
Naples Score	0.62	0.57–0.66	0.45	0.74	< 0.001	
Established risk factors	0.78	0.74–0.83	0.84	0.60	< 0.001	*Z* = 3.37 *p* < 0.001
Established risk factors plus	0.81	0.77–0.85	0.86	0.64	< 0.001	
Naples Score						

Abbreviation: AF, atrial fibrillation; ER, early recurrence; LAD, left atrial diameter.

Figure 4ROC curve for the Naples Score (a), risk factors (b), and established risk factors, including AF duration, LAD, ER, and AF type (c).

**Figure:**
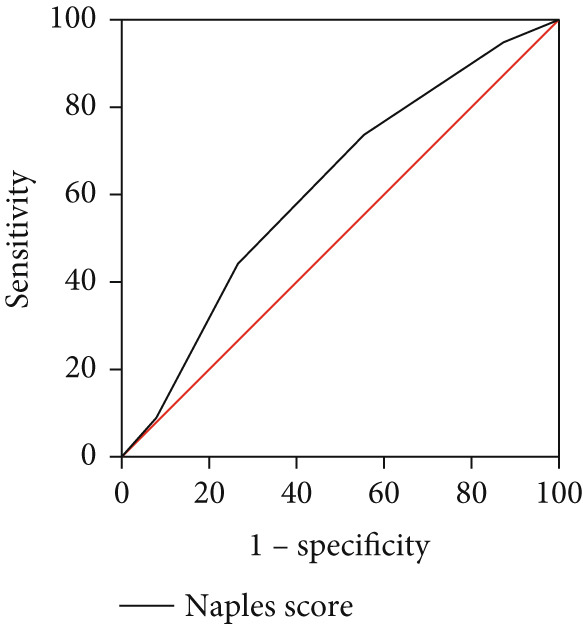
(a)

**Figure:**
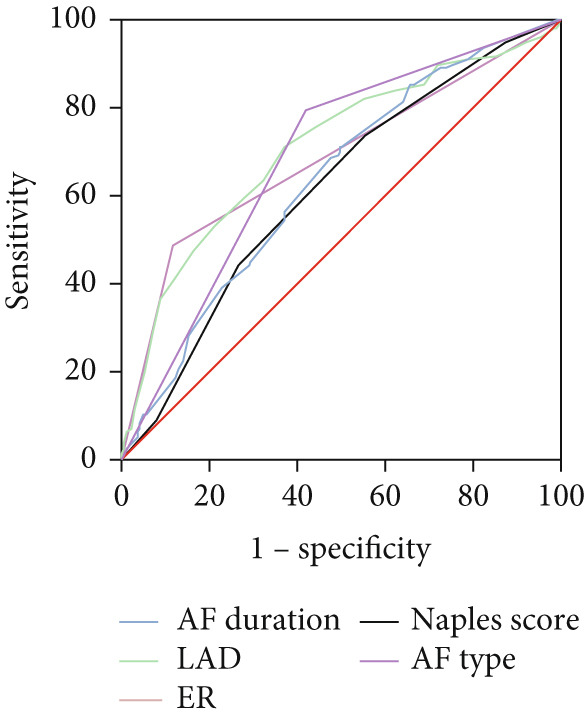
(b)

**Figure:**
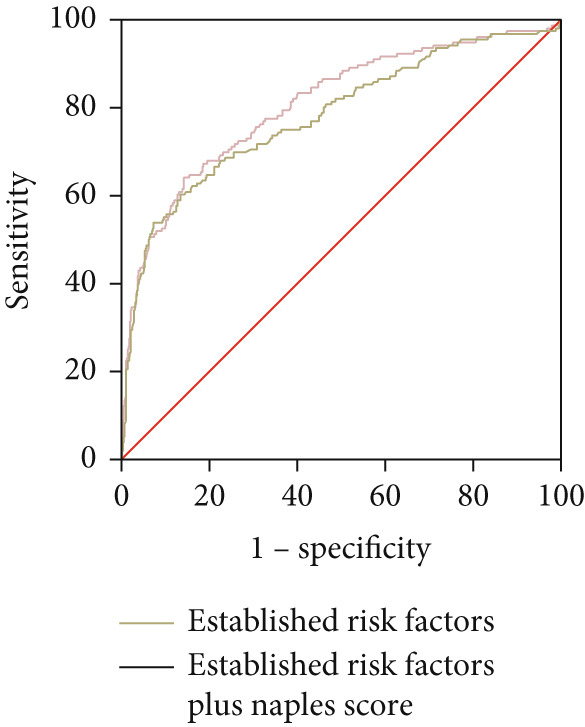
(c)

### 3.3. Kaplan–Meier Survival Curve Analysis of AF Recurrence

**Figure 5:**
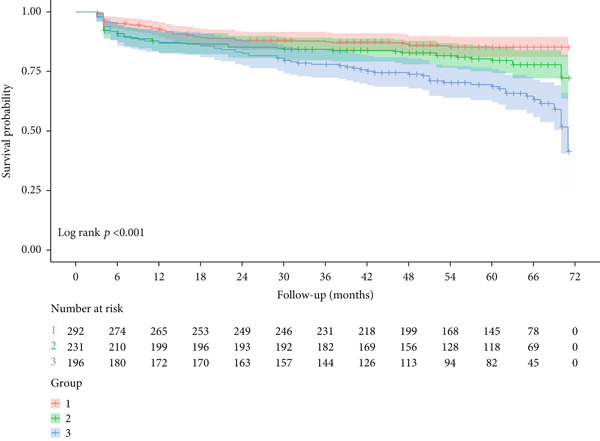
The Kaplan–Meier survival analysis of different Naples Score groups.

The Kaplan–Meier survival analysis revealed significant differences in recurrence after RFCA of AF among different Naples Score groups (Log − rank *p* < 0.001). See Figure 5.

## 4. Discussion

To the best of our knowledge, this is the first cohort study to evaluate the relationship between the Naples Score and the recurrence of AF. The main findings of the study are as follows: the Naples Score is an independent risk factor for AF recurrence following RFCA. Significant differences were observed in the recurrence rates after RFCA among AF patients with varying Naples Scores. Compared with patients with a low Naples Score, those with a high Naples Score were more likely to experience a relapse after RFCA.

AF is the most prevalent arrhythmia encountered in clinical practice, and its incidence has been rising over the past few decades [11]. It is estimated that the global prevalence rate reached 50 million in 2020 [12]. AF can lead to serious complications, including stroke, myocardial infarction, and sudden death [13]. Risk factors for AF include pathological aging, obesity, diabetes, hypertension, and other related diseases. The primary goals of treating AF are to regulate heart rate, restore SR, and reduce the risk of stroke [14]. RFCA is a catheter‐based procedure designed to isolate and potentially eliminate the abnormal electrical lesions that cause AF. For both persistent and PAF, AF ablation is more effective than antiarrhythmic medications. Additionally, the early implementation of rhythm control strategies is a crucial factor in enhancing the success rate of AF ablation [15, 16].

Inflammation may lead to AF, but AF can also promote inflammation, creating a vicious cycle. There is increasing evidence that inflammatory mediators contribute to atrial structural and electrical remodeling. Potential mechanisms include atrial fibrosis, regulation of gap junctions, and abnormal intracellular calcium handling. These abnormalities increase atrial ectopic activity and slow atrial conduction, impairing impulse propagation and promoting refractoriness [17]. Inflammation may play a crucial role in the pathophysiology and prognostic outcomes of AF. Markers of systemic inflammation are significantly associated with atrial remodeling in patients with AF [18]. Research has shown that inflammatory cytokines in cardiac tissue can cause oxidative damage to the atrial myocardium, thereby promoting the occurrence of AF [5]. Serum albumin and TC levels are widely recognized as key indicators of an organism′s nutritional status. Albumin serves not only as a marker of nutritional health but also as an anti‐inflammatory agent that neutralizes free radicals. Additionally, albumin is considered a negative acute phase reactant. It possesses antioxidant and anti‐inflammatory properties, inhibits platelet aggregation and activation, and consequently influences plasma viscosity. Low levels of albumin are associated with an increased risk of mortality and thrombosis in patients with various conditions [19, 20]. NLR is a biomarker of the systemic inflammatory response, and its elevation is closely linked to endothelial cell injury. A previous study has demonstrated that NLR is significantly associated with recurrence after RFCA of AF [9]. LMR is a crucial marker of immune system balance. Low levels of LMR may indicate an imbalance between lymphocytes and monocytes, which can lead to excessive inflammation and an overactive immune response [21]. It has been reported that T lymphocytes play a role in the chronic inflammatory process, which may influence the onset and progression of AF [22]. NLR and LMR are independent predictors of both in‐hospital and long‐term prognosis in patients with ischemic stroke complicated by AF [23]. Yu et al. demonstrated that a lower LMR was associated with a higher 1‐year mortality risk in patients with AF. LMR may serve as a potential prognostic indicator of all‐cause mortality in critically ill AF patients [24]. Our previous study has found that the (hemoglobin, albumin, lymphocyte, and platelet) HALP index is significantly associated with recurrence after RFCA for persistent AF [10].

The Naples Score is a novel indicator of nutritional and inflammatory status, originally developed to predict outcomes in colorectal surgery. The Naples Score is based on levels of albumin and cholesterol, LMR, and NLR [25]. Previous studies have largely confirmed that the Naples Score is significantly correlated with the prognosis of patients with tumors [26, 27]. In recent years, an increasing number of studies have confirmed the diagnostic value of the Naples Score in cardiovascular disease. Altunova et al. demonstrated a strong correlation between the Naples Score and mortality from infective endocarditis (IE), indicating its potential use in predicting prognosis in IE [28]. A cross‐sectional study demonstrated that the Naples Score is significantly associated with the prognosis of patients with heart failure [29]. It has also been found that there is a positive correlation between the Naples Score and cardiovascular mortality rates [30]. Erdogan et al. found that the Naples Score significantly influenced in‐hospital mortality and morbidity in patients undergoing percutaneous coronary intervention [31]. Demirci et al. confirmed that the Naples Score is associated with the long‐term prognosis of patients following transcatheter aortic valve replacement [32]. However, there are relatively few studies on the application of the Naples Score in patients with AF. A recent study has confirmed that a high Naples Score is closely associated with an increased risk of AF following cardiac surgery [33]. Our study confirmed that the Naples Score is an independent risk factor for AF recurrence following RFCA. Significant differences were observed in the recurrence rates after RFCA among AF patients with varying Naples Scores. Patients with a high Naples Score were more likely to experience recurrence after RFCA compared with those with a low Naples Score. Specifically, patients with a high Naples Score were 2.4 times more likely to have a postoperative recurrence than their low Naples Score. ROC curves demonstrated that the Naples Score possesses strong predictive value for recurrence after RFCA for AF. Furthermore, incorporating the Naples Score into the established risk factor model resulted in a significant increase in the AUC (0.81 vs. 0.78, *p* < 0.001).

This study is a retrospective observational analysis with a limited sample size, which may introduce bias in the results. Consequently, large‐scale randomized controlled trials are necessary to confirm the repeatability and generalizability of the findings. Additionally, the absence of standardized methods for detecting arrhythmias, such as long‐term ambulatory electrocardiography, may result in an underestimation of AF recurrence.

## 5. Conclusion

Our study demonstrated that the Naples Score is a significant independent predictor of AF recurrence following RFCA. Compared with patients with a low Naples Score, those with a high Naples Score were more likely to experience a relapse after RFCA.

## Ethics Statement

The study was conducted in accordance with the Declaration of Helsinki and received approval from the Ethics Committee of Yantai Yuhuangding Hospital of Qingdao University. All patients provided informed consent.

## Disclosure

All authors reviewed and approved the final manuscript.

## Conflicts of Interest

The authors declare no conflicts of interest.

## Author Contributions

Zhen Wang and Chunxiao Wang contributed to the conception and design of the study. Yilin Qu and Hua Wang collected the data. Zhen Wang and Yilin Qu wrote the manuscript and conducted the analysis, engaging in constructive discussions throughout the process.

## Funding

This research was supported by funds from the National Natural Science Foundation of China, No. 82200503.

## Data Availability

The datasets utilized and analyzed in this study are available from the corresponding author.
